# National and Transnational Security Implications of Asymmetric Access to and Use of Biological Data

**DOI:** 10.3389/fbioe.2019.00021

**Published:** 2019-02-25

**Authors:** Kavita M. Berger, Phyllis A. Schneck

**Affiliations:** ^1^Gryphon Scientific, LLC, Takoma Park, MD, United States; ^2^Promontory Financial Group, an IBM Company, Washington, DC, United States

**Keywords:** biotechnology, cybersecurity, information security, data vulnerability, biological data, biosecurity, data access, data protection

## Abstract

Biology and biotechnology have changed dramatically during the past 20 years, in part because of increases in computational capabilities and use of engineering principles to study biology. The advances in supercomputing, data storage capacity, and cloud platforms enable scientists throughout the world to generate, analyze, share, and store vast amounts of data, some of which are biological and much of which may be used to understand the human condition, agricultural systems, evolution, and environmental ecosystems. These advances and applications have enabled: (1) the emergence of data science, which involves the development of new algorithms to analyze and visualize data; and (2) the use of engineering approaches to manipulate or create new biological organisms that have specific functions, such as production of industrial chemical precursors and development of environmental bio-based sensors. Several biological sciences fields harness the capabilities of computer, data, and engineering sciences, including synthetic biology, precision medicine, precision agriculture, and systems biology. These advances and applications are not limited to one country. This capability has economic and physical consequences, but is vulnerable to unauthorized intervention. Healthcare and genomic information of patients, information about pharmaceutical and biotechnology products in development, and results of scientific research have been stolen by state and non-state actors through infiltration of databases and computer systems containing this information. Countries have developed their own policies for governing data generation, access, and sharing with foreign entities, resulting in asymmetry of data sharing. This paper describes security implications of asymmetric access to and use of biological data.

## Introduction

Advances in computer science, engineering, and data science have changed research, development, and application of biology and biotechnology in the United States and internationally. Examples of changes include: (a) increased reliance on internet connectivity for research and laboratory operations (Accenture, 2015; Bajema et al., 2018; Olena, 2018); (b) increased use of automation in life-science laboratories (Chapman, 2003); (c) application of the “design-build-test” paradigm to create new biological organisms (Agapakis, 2014; Carbonell et al., 2018); (d) increased generation, analyses, and computational modeling of information about biological systems, cells, and molecules (Thurow et al., 2004; Walpole et al., 2013); (e) treatment of organisms and DNA as materials rather than phenomena to study (Service, 2017; Anderson et al., 2018; Patel, 2018); and (f) new funders such as venture capital, crowdfunding platforms, and foreign companies and governments (Von Krogh et al., 2012; Cha, 2015; Mervis, 2017). These changes have transformed the scientific, agricultural, and health communities' ability to understand and manipulate the world around them. In addition, the changes have enabled an influx of new practitioners and problem-solvers into biology, providing opportunities for education and research all over the world.

Biotechnology harnesses the capabilities of computer, data, and engineering sciences to establish and advance new fields such as synthetic biology, precision medicine, precision agriculture, and systems biology. Cloud-based platforms and open source, easy-to-use software enable scientists from anywhere in the world to use advanced data analytics in their studies. The software and hardware emerging from these fields improve our collective understanding of molecular and systems-level genetics, new drug therapies for longer and better quality of life, and design of novel and/or unnatural organisms. Critical to these pursuits is the sharing of research results and underlying data, without which societal decision-making about human, animal, plant, and environmental health cannot be realized fully. However, during the past two decades, concerns about data sharing have been raised, resulting in the issuance of international, regional, and national-level policies governing access to different types of data, including biological data. In addition, the platforms through which data are stored, transported, and analyzed may be vulnerable to unauthorized acquisition of information by malicious actors, which could lead to significant economic and physical harms to the health, safety, and security of a population. Although not considered “dual use life sciences research of concern” U. S. Government, 2012, 2014), the potential for both benefit and risk to humanity meets the spirit of the dual use concept (National Research Council, 2004). Given the significant benefits afforded by data sharing and analysis, this paper highlights current data protection policies, potential risks of data exploitation by malicious actors, and potential strategies to mitigate those risks and promote rapid recovery in biotechnology fields that are breached.

The interconnectedness between the digital and biological worlds can be exploited by state actors, malicious nonstate actors, and hackers through a variety of means, resulting in harmful consequences from potential theft of information, promulgation of incorrect information, and/or disruption of activities (Lord and Forbes Technology Council, 2017; Souza, 2018; Ward, 2018). For example, theft of proprietary information from a pharmaceutical or biotechnology company may reveal trade secrets and allow competitors to develop superior products and/or bring existing products to market more quickly (Friedman, 2013), stifling innovation in the global commercial market and allowing adversaries to create harmful, untested therapies. Another example is theft of hundreds of millions of electronic healthcare records, the uses of which are not clear (Bogle, 2018; Cohen, 2018; Healthare IT News Staff, 2018; Huang and Steger, 2018; Keown, 2018). Although unauthorized access to protected data may be aided by technical vulnerabilities in networked computer systems, poor security practices, insider threats in academia, industry, and health facilities, and legal business dealings also can enable adversary access to such data (Lynch, 2017; Rappeport, 2018; South China Morning Post, 2018; Zhu, 2018). For examples, more than half of all data breaches at healthcare facilities are caused by healthcare personnel errors, a quarter of which resulted in unauthorized access to or disclosure of patient records through sharing of unencrypted information, sending information to the wrong patients, and accessing the data without authorization (Bai et al., 2017; Michigan State University, 2018). In addition, the Federal Bureau of Investigation (FBI) has raised national security concerns about foreign access to genomic data of U.S. citizens through legitimate scientific collaboration, funding of scientific research, investment in genomic sequencing companies [e.g., China-based WuXi Healthcare Ventures investment in the U.S.-based 23andMe (Biospace, 2015; Mui, 2016)], and purchase of companies (e.g., Complete Genomics) (Baker, 2012; GenomeWeb, 2012). As vulnerabilities are created through scientific advances, such as the use of machine learning algorithms to trick fingerprint authentication systems, new risks are identified (Bontrager et al., 2018; Nyu Tandon School of Engineering, 2018). Some of these concerns have resulted in the passage of the 2018 Foreign Investment Risk Review Modernization Act, which has initiated reform of the U.S. Government process for evaluating foreign investment in U.S. entities and export control of emerging technologies (Rappeport, 2018; U.S. Congress, 2018). Yet, these policy activities largely are reactive, rather than proactive.

## Current Approaches for Protecting Data

Preventing accidental and deliberate risks typically involves the use of cyber and information security systems that include technological and behavioral solutions. Protection of laboratory control systems, computer networks, and databases often involves the use of technological solutions. However, some risks are addressed better through training of personnel to recognize and report phishing attempts, ensure sensitive information is encrypted, and prevent unauthorized individuals from gaining access to sensitive data, databases, and computer networks. To enhance security, policies for promulgating these practices for specific materials and information have been issued. For example, the U.S. Biological Select Agents and Toxins Regulations include guidance for network security to prevent failure of laboratories, equipment, and access controls to facilities and data (Federal Select Agent Program, 2017). In addition, the U.S. has policies for protecting individual privacy, several of which were described in a 2014 report sponsored by the White House (Podesta et al., 2014). However, error, carelessness, or negligence by personnel can counteract the benefits afforded by security measures and may lead to devastating consequences if biological data and materials are involved.

Although policies for protecting biological data from cyberattack are limited, policies that govern data access and sharing are prevalent. These top-down, data access policies intend to protect individual rights and/or prevent sharing or distribution of data, including biological data. Examples of recent policies include: (a) the 2018 update of the European Union General Data Protection Regulation (European Commission, 2018), which strengthened the European Union's rules for protecting personal data of individuals, in part by giving its citizens “more control over their personal data;” (b) the 2018 Chinese Personal Information Security Specification, which is one system under the Chinese Cybersecurity law, involves the “collection, storage, use, sharing, transfer, and disclosure of personal information,” and enables companies operating in China to access data to “not hamper the development of fields like AI” (Sacks, 2018); (c) the 2018 General Data Protection Law in Brazil, which provides a framework for the use of personal data in Brazil (Soares, 2018); and (d) the U.S. Health Insurance Portability and Accountability Act of 1996 (HIPAA), which promotes the protection of privacy and security of patient health information in the United States (Department of Health and Human Services, 2017). At the same time, the U.S. has issued policies governing data generation, access, and sharing to promote information-sharing and transparency of government-sponsored research (Noorden, 2013). Internationally, the Nagoya Protocol of the Convention on Biodiversity^1^ promotes governance on access to and fair, equitable sharing of the benefits from the use of non-human biological data. However, questions exist about whether the Nagoya Protocol focuses more on biological samples that provide genetic information or the genetic information itself, which ultimately affects national-level efforts for codifying the international agreement (Dos et al., 2018). Despite these activities, protection of some data, such as personal health data, may not extend beyond a country's borders and may apply only to data collected by certain entities. Furthermore, data protection polices do not extend to information that already has been stolen. Taken together, these national, regional, and international level policies for data protection may not prevent the inappropriate or unauthorized acquisition of data to different actors, the consequences of which are unclear for biotechnology data.

## Vulnerability of Biotechnology Data

The primary challenges in identifying, assessing, and mitigating security vulnerabilities of biotechnology data are understanding: (a) how the data may be exploited by adversaries and what consequences result from this exploitation; and (2) what potential negative effects may arise from digitalization of biotechnology and advanced computation of biological data (Bajema et al., 2018). The term “biotechnology” refers to the exploitation of biological processes for industrial and scientific purposes, and includes genetic manipulation of microbes, plants, animals, human cells, nucleic acids (the building blocks of genomes), and proteins (the functional units in cells). This definition is expanded further to include generation, incorporation, and use of digital forms of biological data. These biological data may be available online through databases, such as the U.S. National Center for Biotechnology Information's GenBank^2^, or generated in a laboratory and stored, shared, and/or analyzed locally or remotely (via online and/or cloud-based software). By attempting to answer the questions posed above, specific risks associated with the legal and illegal acquisition of biological data may be identified and mitigated.

Although extraordinary advances in computing power are enabling unprecedented scientific discoveries, its application to biology and healthcare is increasing without effective protection from the risks of adversary acquisition or accidental misuse of information. Scientific data that is generated in basic and applied research laboratories in academia, non-profit research organizations, service providers, and some industry research facilities may be considered fundamental research destined for publication and public benefit. These data are not necessarily sensitive, but they do represent the results of significant investment by governments, industry, investors, and philanthropic organizations. Therefore, theft or large-scale acquisition of these data may have adverse economic consequences to the organization, field, or nation, especially if acquisition was directed by adversarial nation-states to gain competitive advantage in a given sector (Blair and Huntsman, 2013). As previously described, databases that store sensitive and/or non-sensitive biological data have been infiltrated by external actors and accessed by unauthorized individuals. Although measures to protect data have been implemented in several institutions, cyber and information security policies, practices, and compliance vary across biotechnology sectors, location, and organization type (e.g., academia, industry). Although implementation of cyber, information, and data security in biological facilities can help to minimize the potential for deliberate or accidental release of protected biological data, these measures are insufficient on their own (Press, 2018).

Furthermore, the increasing size and volume of the datasets, and the complexity of analytic technologies has led many scientists to rely on cloud-based platforms to store, transfer, and analyze data. These platforms and technologies, including online analysis software and applications, often do not prevent unauthorized access to data or ensure software fidelity. Although mitigating specific vulnerabilities may be possible on an individual platform or technology level, implementing protections across the various data generation, analysis, transfer, and storage platforms currently in use in academia, industry, government laboratories, and healthcare facilities is challenging. Countering these risks requires the identification of consequences that are of particular concern to public safety and national security, evaluation of vulnerabilities that may enable the realization of these consequences, and identification of measures to address these vulnerabilities.

## Possible Prevention and Mitigation Approaches

Modern cyber and information security reflects the risks experienced as the internet has grown and diversified, and as the capabilities for and speed of storing, processing, and transporting information have increased exponentially (Denning and Lewis, 2017). The internet was built without a priority on the protection of data whether “at rest” (i.e., stored data) or “in motion” (i.e., data in transit) (Dauch et al., 2009; Inap, 2013). Current strategies for addressing cyber risks focus on remediation through regulation, organizational support, and actions taken by data owners and consumers in the form of encryption technologies, access control measures, awareness-raising campaigns, risk assessment, blocking, limiting publication of sensitive information, and other similar practices. The challenge is understanding how these measures are to be applied to biotechnology data, how to balance the cost of implementation with the consequences if left unprotected, and what vulnerabilities cannot be mitigated using commercial products.

Often the entities that assess their cyber vulnerabilities and invest in cyber and information security measures are compelled to do so because of regulation and fiscal responsibility (McDonald, 2017). However, unlike financial information, biotechnology data is regulated in some countries, but not others. For example, China issued a recent policy requiring a domestic collaborator and Ministry-level approval for research involving genomic data of Chinese citizens and/or biological samples obtained in China to prevent exploitation of these data and samples (Tuzman, 2018). This and similar policies raise questions about their intended and unintended effects to nations, to the scientific community, and to international security mainly because the policies that may benefit one country could harm another. These harms may reveal new types of risks associated with the acquisition and use of data to manipulate biological systems. These risks may be perpetrated by different actors; affect sector and country economies, commercial biotechnology, and pharmaceutical markets domestically and internationally; and alter global strategic power dynamics.

The risks associated with biotechnology data do not conform to traditional biosecurity concerns, which focus primarily on risks to human health or the food and agriculture economy. These risks involve multiple domains, sectors, and nations resulting in outcomes such as shifting of balance of power of nations at the international level, which could have downstream effects on areas that overlap with biosecurity interests (e.g., biosafety and biosecurity, biothreat reduction, and global health security). Strategies for bridging the biological, cyber, information, and data security include: (a) collaboration between the biological and cybersecurity communities; (b) end-to-end risk assessments; (c) data-specific risk and vulnerability assessments; and (d) application of the NIST Cybersecurity Framework for protecting biological data.

Formal collaboration between the biotechnology and biological, information, data, and cyber security communities would enhance efforts toward identification of risks and vulnerabilities associated with data management, provenance, and integrity, and risk mitigation strategies. Technologies are readily available to protect data, but their use must be harmonized worldwide, because protecting data in one database is ineffective if another database remains vulnerable to external threats. Furthermore, organizations may evade regulatory requirements and industry standards in protecting data because of perceived lack of cost savings for implementing cybersecurity measures or lack of awareness of the risks, which could lead to investor, intruder, or adversary access to sensitive information that may be stored in databases or transferred between computers. These vulnerabilities may be exacerbated by limitations of national laws to other sovereign states, and differences in interpretation of the types of data included in the scope of existing laws. **Given these potential vulnerabilities, the cybersecurity and biotechnology communities must engage to create best practices and processes to protect data and mitigate risk while reaping the benefits of computing technology applications to biotechnology**.

End-to-end assessments of the data storage, processing, and transport pipeline can identify outstanding vulnerabilities and technical gaps that may be addressed with currently available cyber, information, and data security solutions. This process would enable identification of gaps for which these measures are insufficient and of institutions that are responsible for implementing controls. Without this type of assessment, vulnerabilities may exist along the pipeline without its users' knowledge. A lack of rigorous analysis makes biological data vulnerable to acquisition or alteration by witting adversaries, potentially resulting in theft of intellectual property for commercial gain, foreign government acquisition of genomic data from large portions of a population for undefined purpose or compromise of software and data integrity. At least one country promotes acquisition of data though legitimate commercial practices (e.g., providing sequencing services to customers; partnering with academia, independent research institutions, and universities; and foreign investment), talent promotion programs (Capaccio, 2018; Nature Jobs, 2018), and theft of data (Riley and Walcott, 2015; Dilanian, 2018; Kaiser and Malakoff, 2018; Wilber, 2018). The FBI has expressed concerns about the theft of U.S. genomics and health information through cyberattacks and foreign investment in the U.S. biotechnology industry (You, 2017). The FBI argues that acquisition of this information can give adversaries an unfair advantage in the international pharmaceutical or biotechnology marketplace. Others have expressed concern about questionable use of genetic information that countries obtain from their own citizens or from other countries' citizens (Human Rights Watch, 2017; Lynch, 2017; Pauwels and Vidyarthi, 2017). **These risks could be addressed by conducting an end-to-end risk assessment of the software and equipment involved in the data pipeline within individual organizations, between organizations, and across countries**.

Defining the consequences of greatest concern to national security is an initial step toward assessing the risks and vulnerabilities of the information itself and data-specific risk mitigation strategies. Evaluating these risks enables the identification of content-specific approaches for detecting and countering exploitation of vulnerabilities by insider and external actors. Without these assessments, only generic cyber and information security measures will be implemented. However, these measures are insufficient to counter adversaries who are intent on acquiring data through a variety of technical, social engineering, or other means. Given this reality, rapid detection and resilience (i.e., rapid recovery after a breach) are critical for reaping the benefits and minimizing the vulnerabilities of advanced electronic computation and mass connectivity. In 2014, the White House explored technology needs for protecting the security and privacy of exposed data, including healthcare data (Executive Office of the President, 2014; President's Council of Advisors on Science Technology, 2014). But, these studies did not define consequences of concern related to the unauthorized acquisition of vast amounts of biological data, effectively limiting the identification of data-specific or process-specific prevention measures. **Therefore, risk assessments of specific types of data are equally as important to conduct as analyses of vulnerabilities of laboratory control systems, data management platforms, and computer networks**.

Application of the National Institute of Standards and Technology (NIST) Cybersecurity Framework to all systems of storage, processing and transport of biological data would help explore where, how, and by whom data is processed with the goal of protecting valuable scientific and health information (National Institute of Standards Technology, 2018). The NIST framework involves a collaboration of private sector and government cybersecurity experts that seek to apply the five principles of data protection (i.e., identify, protect, detect, respond, and recover) to systems, including those on which biological data are generated, processed and transported. The framework could augment existing or newly-implemented efforts of vulnerability detection and mitigation, thus decreasing unauthorized exposure of sensitive data. The NIST framework is a widely accepted paradigm for cyber risk management and best practices (Department of Homeland Security, 2018; Lohrmann, 2018; Roncevich, 2018). In the U.S., this framework has been used in regulatory dialogues to demonstrate rigor toward cybersecurity in sectors for which such requirements are not well-documented in law. **Application of the NIST framework to biotechnology can enhance data protection and a focus on rapid detection of nefarious activity and resiliency after an attack**.

These suggestions describe various approaches toward protecting biological data from unauthorized acquisition and use, enhancing efforts to preserve data integrity and provenance, and enabling future benefit of biotechnological advances.

## Author Contributions

KB and PS contributed equally to this manuscript. The concepts, conclusions, and recommendations were generated jointly by the authors and built on their respective expertise in the biological sciences and biosecurity, and computer science and cybersecurity.

### Conflict of Interest Statement

KB was employed by Gryphon Scientific. PS was employed by Promontory Financial Group, which is an IBM Company. The authors declare that the paper was written in the absence of any commercial or financial relationships that would constitute a conflict of interest.

## Footnotes

^1^Convention on Biodiversity. About the Nagoya Protocol. Available online at: https://www.cbd.int/abs/about/ (Accessed November 23, 2018).

^2^National Center for Biotechnology Information. GenBank. Available online at: https://www.ncbi.nlm.nih.gov/genbank/ (Accessed November 23, 2018).
